# Addition of *Pachira aquatica* oil and *Platonia insignis* almond in cookies: Physicochemical and sensorial aspects

**DOI:** 10.1002/fsn3.1368

**Published:** 2020-05-27

**Authors:** Tania G. Silva, Marcia G. C. Kasemodel, Otávio M. Ferreira, Rogério C. L. da Silva, Clitor J. F. Souza, Eliana J. Sanjinez‐Argandona

**Affiliations:** ^1^ Departamento de Engenharia de Alimentos Faculdade Engenharia Universidade Federal da Grande Dourados – UFGD Dourados Brasil; ^2^ Faculdade de Zootecnia e Engenharia de Alimentos Universidade de São Paulo Pirassununga Brasil; ^3^ Laboratório de Química Universidade Estadual do Mato Grosso do Sul Naviraí Brasil

**Keywords:** Amazon fruit, fatty acids profile, functional food, sensorial analysis

## Abstract

This article investigated the use of *Pachira aquatica* (*PA*) fat and *Platonia insignis* (*PI*) nuts as ingredients in the preparation of cookies. Seven formulations containing *PA* fat and/or *PI* nuts were studied by changing the formulation proposed by AACC, and samples were evaluated considering physical–chemical, microbiological, and sensory characteristics. Formulations F1, F4, and F5 showed higher mass loss and lower expansion factor after cooking. Formulations F4, F6, and F7 presented a greater increase in diameter. In turn, formulations F5, F6, and F7 presented greater thickness. The content of fatty acids varied according to the composition of each biscuit, and formulations 2 and 3 presented the best lipid profile (oleic acid ~32%). In addition, it was observed that the addition of *PI* almond increased the fiber content (~7.15%). The sensory evaluation showed that formulation F5 obtained a score of 5, proving that the partial replacement of hydrogenated vegetable fat with *PA* fat and grated coconut with *PI* almond favored the panelists' purchasing decision. The results indicate that unconventional sources of lipids and nuts can be used without loss of quality in biscuits.

## Graphical abstract

Unconventional sources of lipids may enriched cookies. Munguba fat and bacuri nuts are functional source of lipids. Cookies presented good sensorial and physicochemical aspects.

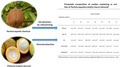

## 1 INTRODUCTION

The *Pachira aquatica* (*PA*) Aubl. (Malvaceae), commonly known as munguba, castanheira, wild cocoa, and maranhão nut, is a native tree of Mexico (Paula, Cruz, & Barbosa, 2006). In Brazil, it is common in the Amazon region and occurs mainly in streams and rivers, from which originated its scientific name. Some studies have shown that *PA* seeds present a high amount of lipids (44%), 12.9% of protein showing a high amount of essential amino acids such as tyrosine, tryptophan, and threonine (De Bruin, Heesterman, & Mills, 1963; Oliveira et al., 2000). Leterme, Buldgen, Estrada, and Londoño (2006) reported that *PA* seeds are an excellent source of phosphate, magnesium, zinc, iron, and copper. Although *PA* readily adapts to very diverse soil conditions and even though its fruits produce almonds that may be consumed naturally, cooked, or roasted by the Amazonian populations (Andrade‐Cetto & Heinrich, 2005), it is not yet commercially exploited.

The bacurizeiro is a plant species that belongs to the Clusiaceae family, subfamily Clusioideae, genus Platonia Mart. It is a monotypic species, classified as *Platonia insignis* (*PI*) Mart, and is popularly known as “bacuri” or “acuri” (de Freitas et al., 2017). The *PI* fruits contain phytochemical constituents with antioxidant properties, such as tannins, flavonoids, anthocyanins, and simple phenols. The *PI* oil contains fatty acids, including palmitic, linoleic, stearic, and oleic acids, and are, therefore, of particular interest to the food industry (Freitas de Lima et al., 2016).In human food, it is interesting to investigate the use of *PA* and *PI* as ingredients in the elaboration of food products that present a high nutritional value while adding value and favoring the preservation of the species. Thus, the insertion of *PA* fat or *PI* almond into products of great acceptability such as cookies may be an opportunity.

Cookies are widely accepted by people of all ages and social classes (Kurek & Wyrwisz, 2015). Formulations mainly include wheat flour, sugar, and hydrogenated fat. Because of their attractive sensory characteristics, the addition or substitution of certain ingredients in cookies is possible. In fact, the addition of other ingredients to cookies is widely performed, whether to incorporate functional constituents such as soluble fibers (Bick, Fogaça, & Storck, 2014; Vitali, Dragojević, & Šebečić, 2009), to replace wheat with flours obtained from unconventional fruits such as defatted sesame seed meal (Clerici, Oliveira, & Nabeshima, 2013), to add pequi, baru or buriti (Pineli et al., 2015; Santos et al., 2011; da Silva, Monteiro, & da Rosa, 2014), among others. The potential role of PA fat and PI almond in the formulation of cereal‐based foods has not been addressed in the scientific literature yet. Therefore, evaluating the use of these functional products as a food ingredient would allow for the development of baked products with improved nutritional value, while valuing and favoring the preservation of the species. Given this promising application, this work aimed to study the effect of the addition of *PA* fat and *PI* almond in the physicochemical, nutritional, and sensory characteristics of cookies.

## 2 MATERIAL AND METHODS

### 2.1 Material

The *PA* and *PI* fruits were randomly collected in the municipality of Dourados—Mato Grosso do Sul (latitude 22°13′16″, longitude 54°48′20″ and altitude of 430 m). The fruits were hygienized and sanitized in sodium dichloroisocyanate solution dihydrate 0.66% (w/w) (active chlorine content 3% w/w) for 10 min. The almonds (endosperm) were removed for posterior use (*PI*) or even for oil extraction (*PA*). In both cases, the almonds were packed in inert plastic containers and then stored at −5°C until use.

Type 1 wheat flour with 10% protein (Dona Benta), corn starch (Nestlé), refined sugar (União), brown sugar (Jasmine), hydrogenated vegetable fat (Bunge), milk (Tirol), eggs (Qualitá), baking powder (Royal), vanilla flavoring (Fleischmann), salt (Sisnei), chocolate (Nestlé), and grated coconut (in natura) were purchased in the local market of Dourados, MS, Brazil (latitude 22°22′03″, longitude 54°77′37″).

### 2.2 Oil extraction

The oil was extracted by cold pressing in an expeller‐type press (Ecirtec ‐ MPE‐40P). For the extraction, the almonds were previously dehydrated at 45°C for 48 hr in an oven with air circulation at a speed of 0.5 m/s. The extracted oil was centrifuged at 4226*g* for 15 min at room temperature, transferred to amber containers and stored under refrigeration (7°C) until use.

### 2.3 Cookie preparation

The cookies were prepared by modifying a method of the AACC (2000), in which wheat flour (60%) and corn starch (40%) accounted for 100%. Milk, eggs, baking powder, and flavoring were incorporated in the same amount in all formulations. The percentage of each ingredient was calculated using the total flour and starch mass, as follows: milk (9%), eggs (9%), baking powder (0.42%), vanilla flavor (0.33%), and salt (0.25%), in addition to the ingredients shown in Table 1.

**Table 1:** Formulation of cookies containing or not fats of *PA* and/or *PI* almond

Ingredients % (w/w)	Cookie formulation						
	F1	F2	F3	F4	F5	F6	F7
Refined sugar	43	–	43	21.5	43	21.5	29
Brown sugar	–	43	–	21.5	–	21.5	14
Hydrogenated vegetable fat	–	27	27	13.5	13.5	27	18
*PA* fat	27	–	–	13.5	13.5	–	9
Grated coconut	11	11	–	11	5.5	5.5	7.4
*PI* almond	–	–	11	–	5.5	5.5	3.6

The preparation of the cookies consisted of mixing the moist ingredients in a domestic shaker (300*g*) until a cream was obtained, and then, the dry ingredients were incorporated and mixed until a homogeneous mass was obtained. Chocolate pieces (66.66% w/w) were added in all formulations. The cookies were shaped in the form of 30 mm diameter and 5 mm thick disks and baked at 200°C for 15 min in a semi‐industrial oven (Venâncio—Fc4emv). They were then cooled to room temperature (25°C) and conditioned in hermetically sealed containers until the time of analysis.

### 2.4 Physicochemical analysis

The mass of the cookies was determined in a semianalytical balance (BEL Engineering) before and after baking. The diameter and thickness were determined with a digital caliper (Digimess). The expansion factor was calculated by the ratio between the diameter and the thickness of the cookies after baking (AACC method 10‐50D, 2000). The specific volume was determined by the kernel seed displacement method (Moraes, Zavareze, Miranda, Salas‐Mellado, 2010), and the yield was calculated from the difference between the final (after baking) and the initial (before baking) mass of the cookies, expressed as a percentage.

The color analysis was performed using a Konica Minolta (Model CR‐400/Cr‐410) colorimeter, with a D65 illuminator, a 10° Angle observation and a CIE L* a* b* system (Commission Internationale de L'Eclairage, 1986). The parameters evaluated were as follows: Luminosity (L*) ranging from 0 (black) to 100 (white), chromaticity of red‐green (a*) and blue‐yellow (b*), hue angle (°h), and color saturation (*C*).

The instrumental texture analysis was performed by the Texture Analyzer TA XT‐2 (Texture Technologies Corporation), with an HDP/WBR (Blade Warner Bratzler & reversible blade) rectangular steel blade under the following conditions: pre‐test speed 1, 0 mm/s, 3.0 mm/s test, and post‐test speed 10.0 mm/s, 10 mm rupture distance and 10 g contact force.

All physical analyses were performed randomly for each batch of the different formulations made.

### 2.5 Proximate composition

Moisture determinations were carried out in a greenhouse at 105°C (AOAC, 1995), lipids were determined by the cold method (Bligh & Dyer, 1959), fixed mineral residue was determined by muffle incineration at 550°C, protein was determined by the micro‐Kjeldahl method (Labtec DT2008), and dietary fiber was determined according to the method proposed by AOAC (1995). Carbohydrates were calculated by the difference of the other components. The total energy value (TEV) was calculated using the following Atwater conversion factors: 4 kcal/g proteins, 9 kcal/g lipids, and 4 kcal/g carbohydrates (Merrill & Watt, 1955).

### 2.6 Fatty acid composition

Total lipids were extracted from the samples using the Bligh and Dyer method (1959). For the transesterification of the triglycerides, approximately 50 mg of the sample was transferred to 15 ml falcon‐type tubes, in which 2 ml of heptane was added. The mixture was subsequently stirred until complete dissolution of the grease material, and then, 2 ml of KOH (2 M) in methanol was added. The mixture was stirred for approximately 5 min, and after phase separation, 1 ml of the higher phase (heptane and fatty acid methyl esters) was transferred to 1.5 ml Eppendorf flasks. These flasks were hermetically sealed and stored in a freezer at −18°C for further chromatographic analysis.

The fatty acid composition was determined by gas chromatography (Shimadzu, GC‐2010 Plus Capillary GC) using gas chromatography with a flame ionization detector. For elution, a 100 m × 0.25 mm × 0.20 μm molten silica capillary column was employed. The oven temperature was programmed to start at 100°C and was held for 1 min and then raised to 170°C at 6.5°C/min. Subsequently, another elevation of 170–215°C was performed at 2.75°C/min, and the temperature was maintained for 12 min. Finally, a final elevation was performed from 215 to 230°C at 40°C/min. The injector and detector temperatures were 270 and 280°C, respectively. The 0.5 μl samples were injected in split mode, using nitrogen as a carrier gas with a drag speed of 1 ml/min. The identification of the fatty acid methyl esters was performed by comparison to the retention times of the sample compound with the standards (Sigma) eluted under the same conditions as the sample.

### 2.7 Sensory analysis

A nine‐point hedonic scale was used for sensory analysis, anchored at extremes 1 (dislikes extremely) and 9 (likes extremely). The attributes analyzed were aroma, color, taste, sweetness, crispness, and global acceptance. The purchase intention was assessed using a five‐point scale (1 = certainly would not buy and 5 = certainly would buy). Ninety‐six untrained judges of both sexes, aged between 17 and 58, participated in the analysis. The panelists signed the informed consent form before the test, according to Resolution 466/12 of the Ministry of Health (Brasil, 2013). The analysis was performed on different days. In the first step, the samples of four formulations (F1, F2, F3, and F4) were presented to the panelists. In the second step, the samples of formulations F5, F6, and F7 were evaluated, as well as F4, which presented higher acceptance in the first stage of the test. The samples were presented in containers labeled using three‐digit codes and were served monadically with water, in order to clean the taste buds. The sample presentation order was distributed randomly, balanced and randomized in complete blocks. This study was approved by the Research Ethics Committee of the University of Grande Dourados (number of the Certificate of Presentation for Ethical Assessment: 2.702.611).

### 2.8 Microbiological analysis

The *Salmonella* sp., Coliform 45°C and Coagulase‐positive Staphylococcus counts were performed on the cookies with the best formulations, according to the methodology described by Downes, Ito, and American Public Health Association (2001).

### 2.9 Statistical analysis

The StatSoft program (Statistica 8.0) was used for statistical analysis. The data referring to the physicochemical analyses and sensory acceptance tests were subjected to the analysis of variance (ANOVA) by the *F* test and to the comparison of means by the Tukey test (*p* < .05). For the sensory preference ranking test, the difference between treatments was verified by the Newell and MacFarlane table (*p* < .05).

## 3 RESULTS AND DISCUSSION

### 3.1 Proximate composition

The cookies were prepared by modifying the AACC method (AOAC, 1995), and the results of the proximal composition are shown in Table 2. The moisture content varied from 4.38 to 6.19 g/100 g, which is in the range of commercial cookies that typically present a moisture content that varies from 1 to 6 g/100 g. In general, cookies are considered very‐low moisture content products and it is important to highlight that the moisture content can contribute to the texture, sensorial and microbial properties of the product, thus indicating the quality and shelf life (Ng, Robert, Wan Ahmad, & Wan Ishak, 2017).

**Table 2:** Proximate composition of cookies containing or not fats of *PA* and/or *PI* almond

Constituents (g/100 g)	Cookie formulation						
	F1	F2	F3	F4	F5	F6	F7
Moisture	4.82 ± 0.02^c^	6.19 ± 0.21^a^	4.38 ± 0.02^d^	5.70 ± 0.02^b^	4.53 ± 0.18^d^	4.41 ± 0.08^d^	4.46 ± 0.05^d^
Crude fat	36.53 ± 0.74^c^	40.81 ± 0.23^b^	42.39 ± 0.14^a^	32.48 ± 0.69^d^	33.52 ± 0.79^d^	36.42 ± 0.12^c^	33.20 ± 0.48^d^
Ash	1.16 ± 0.07^e^	1.26 ± 0.1^d^	1.89 ± 0.02^a^	1.58 ± 0.07^b^	1.49 ± 0.04^b^	1.42 ± 0.02^b^	1.34 ± 0.03^c^
Crude protein	6.78 ± 0.22^b^	7.35 ± 0.28^a,b^	6.89 ± 0.43^a,b^	6.98 ± 0.11^b^	6.85 ± 0.19^b^	6.87 ± 0.11^b^	7.71 ± 0.3^a^
Carbohydrate	50.01 ± 0.20^c^	44.4 ± 0.32^e^	44.45 ± 0.47^e^	53.26 ± 0.65^a^	46.49 ± 0.34^d^	50.88 ± 0.51^b^	53.3 ± 0.19^a^

Note

Different letters in each column indicate statistical significant differences in mean values (*p* < .05).

The brown sugar had a significant influence (*p* ≥ .05) on the higher retention of moisture in the cookies made with 100% of this sugar (F2). The addition of sugars in all formulations aided in the softness and the volume of the cookie, in addition to providing a sweet flavor, as well as color and aroma to the product (Kawai, Toh, & Hagura, 2014; Walker, Seetharaman, & Goldstein, 2012). Cookies of the F2 and F3 formulations had a higher amount of lipids, due to the addition of hydrogenated fat, coconut, and *PI* almond. Similar values (30.05%–35.81%) were obtained for cookies made with the addition of sesame and green banana seeds (Agu & Okoli, 2014).

The addition of *PI* almond influenced the increase of the fiber content. The cookies of the F5 formulation presented 0.6% of soluble fiber and 6.86% of insoluble fiber, totalizing 7.15% of dietary fiber. Therefore, the *PA* and *PI* cookies can be considered products with high‐fiber content. From a nutritional standpoint, to claim that a food is a “source of dietary fiber,” it should contain at least 3 g/100 g of serving of total dietary fiber, whereas the claim “high in dietary fiber” is assigned to food categories with at least 6 g/100 g (Fuller, Beck, Salman, & Tapsell, 2016). The importance of dietary fiber intake in human consumption is due to the benefits already evidenced by several studies in the prevention of hypertension, diabetes, obesity, gastrointestinal diseases, and among others (Westenbrink, Brunt, & van der Kamp, 2013).

### 3.2 Technological characteristics

The physicochemical analysis was used to characterize the cookies, and the results are presented in Table 3. In all formulations, the cookies presented a lower mass after baking, which was already expected due to the loss of water. The F1, F4, and F5 cookies presented higher mass loss, but there was no significant difference (*p* ≥ .05). The F5 cookies presented higher diameter and expansion factor after baking (5.29 mm and 2.98%, respectively); however, there was no significant change in thickness when compared to the other cookies (*p* ≥ .05). This can be justified by the presence of fat added to the dough, which interferes with the gluten expansion during the baking process, forming a porous texture, which in turn favors the evaporation of liquids, thus guaranteeing the quality of the texture of the cookies (Jacob & Leelavathi, 2007).

**Table 3:** Physical properties of the different formulations of cookies containing or not fats of *PA* and/or *PI* almond

Physical properties of cookies	Cookie formulation						
	F1	F2	F3	F4	F5	F6	F7
WBB (g)	6.44 ± 0.39^a^	6.56 ± 0.34^a^	6.68 ± 0.25^a^	6.54 ± 0.14^a^	6.52 ± 0.22^a^	6.62 ± 0.23^a^	6.68 ± 0.15^a^
DBB (mm)	29.56 ± 0.02^a^	30.25 ± 0.37^b^	30.30 ± 0.31^b^	30.57 ± 0.52^b^	30.83 ± 0.22^b^	30.89 ± 0.17^b^	30.65 ± 0.42^b^
WAB (g)	5.43 ± 0.32^a^	5.60 ± 0.33^a^	5.72 ± 0.12^a^	5.47 ± 0.02^a^	5.39 ± 0.20^a^	5.67 ± 0.25^a^	5.69 ± 0.02^a^
DAB (mm)	30.40 ± 0.20^a^	31.19 ± 0.44^b^	30.80 ± 0.07^b^	31.70 ± 0.53^b^	31.78 ± 0.24^b^	31.98 ± 0.16^b^	31.80 ± 0.51^b^
Thickness (mm)	5.07 ± 0.02^b^	5.16 ± 0.05^b^	5.06 ± 0.01^a^	5.18 ± 0.03^b^	5.29 ± 0.06^c^	5.32 ± 0.05^c^	5.20 ± 0.01^c^
EF	6.00 ± 0.02^a^	6.05 ± 0.09^a^	6.09 ± 0.02^b^	5.95 ± 0.09^a^	6.00 ± 0.08^a^	6.17 ± 0.02^b^	6.12 ± 0.10^b^
SV (ml/g)	5.60 ± 0.35^a^	5.57 ± 0.34^a^	5.90 ± 0.23^a^	5.79 ± 0.12^a^	5.38 ± 0.15^a^	5.84 ± 0.28^a^	5.59 ± 0.23^a^
WL (%)	15.68 ± 0.35^a^	14.63 ± 0.33^b^	14.37 ± 0.18^c^	16.36 ± 0.16^d^	17.33 ± 0.21^e^	14.35 ± 0.24^c^	14.82 ± 0.08^f^

Note

Different letters in each column indicate statistical significant differences in mean values (p < .05).

Abbreviations: DAB, Diameter after baking; DBB, Diameter before baking; EF, Expansion factor; SV, Specific volume; WAB, Weight after baking; WBB, Weight before baking; WL, Mass Loss.

The largest diameter increase was found in the formulations with brown sugar (F4, F6, and F7). The F5, F6, and F7 formulations presented greater thickness. The substitution of the coarse sugar by the brown sugar and the grated coconut by the *PI* almond (F6) favored the increase of the expansion factor. The expansion factor is related to the ability of the ingredients to absorb water. Generally, cookies with a high content of insoluble fibers present a lower expansion factor (Fradinho, Nunes, & Raymundo, 2015). The *PA* fat influenced the reduction of the expansion factor as can be observed in the F1, F4, and F5 formulations. According to Gaines (1993), the diameter and the expansion factor in cookies may be used to predict the quality of the products, since these factors influence the size of the sample presenting variability, which hinders the standardization of the product.

The cookies with the addition of *PA* and *PI* almond (F5) presented a lower volume. As reported by Armbrister and Setser (1994), the interaction of sugars with fat substitutes can produce cookies with less expansion and, consequently, with lower specific volume and greater softness. However, the specific volume variation is lower, which favors product standardization.

Color is a fundamental attribute to evaluate food quality and visual analysis is the first of the senses to be used in the choice and acceptance of the product, considering the color parameters (Table 4). The F1 formulation cookies had a lighter color (L* = 61.98), due to the addition of coconut and coarse sugar. In comparison, the F2 and F4 formulations were made with brown sugar and presented a darker color (L* = 48.83).

**Table 4:** Color parameters of the different cookies formulations containing or not fats of *PA* and/or *PI* almond

Color	Cookie formulation						
	F1	F2	F3	F4	F5	F6	F7
L*	61.98 ± 1.00^a^	48.83 ± 0.06^b^	53.32 ± 0.16^c^	48.17 ± 0.06^b^	58.63 ± 0.12^d^	53.63 ± 0.20^c^	58.75 ± 0.40^d^
a*	7.09 ± 0.26^a^	7.66 ± 0.02^b^	8.56 ± 0.08^c^	8.66 ± 0.03^c^	6.96 ± 0.02^a^	6.23 ± 0.02^a^	7.63 ± 0.02^b^
b*	19.86 ± 0.04^a^	19.98 ± 0.02^a^	19.97 ± 0.03^a^	17.42 ± 0.03^b^	19.95 ± 0.01^a^	19.44 ± 0.96^a^	20.56 ± 0.25^a^
*C*	21.08 ± 0.61^a^	21.40 ± 0.02^a^	21.73 ± 0.05^a^	19.45 ± 0.034^b^	21.13 ± 0.01^a^	20.41 ± 0.91^b^	21.93 ± 0.23^a^
*H*	1.23 ± 0.004^a,b^	1.20 ± 0.001^a,b^	1.17 ± 0.003^a^	1.11 ± 0.001^a^	1.24 ± 0.001^a^	1.26 ± 0.015^a^	1.22 ± 0.004^a,b^

Note

Different letters in each column indicate statistical significant differences in mean values (*p* < .05).

The cookies presented predominance of the yellow color (b*), ranging from 17.42 to 20.56. The values of parameter a* can be justified by the caramelization of sugars (Asikin et al., 2014). The color saturation (C*) can be explained by the Maillard reaction. During baking, the carbonyl group (C=O) of the carbohydrates interacts with the amine group (NH_2_) of the amino acids, producing melanoidins, which give color and a baked food characteristic aspect with more defined staining verified by saturation. Higher color saturation (C* = 21.93) was also observed in cookies with higher protein content (F7), although they presented a lower amount of sugars.

In the texture evaluation, the cookies presented different strength results with variation from 3,311.43 ± 688.30 to 5,342.85 ± 501.97 N, suggesting products with different levels of crispness. The F5 formulation (with the addition of 50% of *PA* fat and 50% of *PI* almond) presented greater softness (3,311.43 ± 688.30 N), but was firm and crispy. However, higher values of force at rupture are not necessarily related to a lower acceptance or lower product quality.

### 3.3 Fatty acid profile

Fats and oils are available to the body as fatty acids that play a significant role in human health. Table 5 shows the fatty acid composition of the seven formulations. The F1 formulation contained the highest values of total saturated and monounsaturated fatty acids due to the high content of fatty acids present in *PA* (Oliveira et al., 2000; Yang, 2009). While the F2 formulation, produced only with hydrogenated fat, showed a similar content of total saturated and monounsaturated fatty acids to the F3 (*PI* seed) and F6 (brown sugar and *PI* in ratio 50:50) formulations. This occurred because *PI* seed and hydrogenated fat are also rich in saturated fatty acids. The saturated fat content in cookies is related to different beneficial technological properties, such as the ease of handling of the dough during manufacture and the desired crunchiness of the cookies (Tarancón, Salvador, & Sanz, 2013). However, its high consumption is associated with the high risk of developing coronary problems (Ascherio et al., 2009), and according to international dietary claims (EFSA Panel on Dietetic Products, Nutrition, and Allergies (NDA), 2010), consumption of serum fatty acids should be as low as possible.

**Table 5:** Profile of saturated, monounsaturated, and polyunsaturated fatty acids of the cookies made with the different formulations containing or not fats of *PA* and/or *PI* almond

Fatty acids profile (%)	Cookie formulation						
	F1	F2	F3	F4	F5	F6	F7
C8:0	1.07 ± 0.19^a^	1.05 ± 0.02^a^	0.58 ± 0.01^b^	1.15 ± 0.04^a^	1.01 ± 0.04^a^	0.90 ± 0.14^b^	0.69 ± 0.05^b^
C10:0	1.00 ± 0.03^a^	1.01 ± 0.02^a^	0.77 ± 0.08^b^	1.11 ± 0.02^a^	1.05 ± 0.03^a^	1.03 ± 0.25^a^	0.78 ± 0.04^b^
C12:0	7.4 ± 0.35^a^	7.23 ± 0.20^a^	4.75 ± 0.29^b^	7.73 ± 0.08^a^	7.20 ± 0.11^a^	5.83 ± 0.66^b^	4.90 ± 0.10^b^
C14:0	4.6 ± 1.09^a^	3.20 ± 0.11^b^	2.46 ± 0.03^b^	3.12 ± 0.35^b^	3.22 ± 0.03^b^	2.85 ± 0.17^b^	2.63 ± 0.06^b^
C16:0	42.5 ± 0.40^a^	18.10 ± 2.9^b^	20.00 ± 0.10^b^	31.73 ± 0.13^c^	32.24 ± 0.12^c^	21.55 ± 0.49^b^	30.86 ± 0.29^c^
C18:0	13.2 ± 0.43^a^	16.84 ± 0.54^b^	16.82 ± 0.23^b^	14.38 ± 0.09^c^	13.04 ± 0.03^a^	18.00 ± 0.26^c^	16.94 ± 0.17^b^
C18:1n‐9 cis (oleic acid)	21.7 ± 0.66^a^	41.22 ± 1.84^b^	43.48 ± 0.37^b^	30.85 ± 0.17^c^	32.07 ± 0.23^c^	40.25 ± 1.23^b^	33.91 ± 0.27^c^
C18:2n‐6 cis (linoleic acid)	8.5 ± 0.17^a^	11.35 ± 0.43^b^	11.14 ± 0.15^b^	9.93 ± 0.11^c^	10.17 ± 0.07^c^	9.59 ± 0.22^c^	9.29 ± 0.13^c^
SFA	69.77 ± 0.41^a^	41.22 ± 1.84^b^	43.48 ± 0.37^b^	30.85 ± 0.17^c^	32.07 ± 0.23^c^	40.25 ± 1.23^b^	33.91 ± 0.27^c^
MUFA	21.7 ± 0.66^a^	7.90 ± 0.63^b^	7.56 ± 0.12^b^	9.87 ± 0.12^c^	9.63 ± 0.36^c^	8.36 ± 0.33^b^	9.47 ± 0.12^c^
PUFA	8.5 ± 0.17^a^	11.35 ± 0.43^b^	11.14 ± 0.15^b^	9.93 ± 0.11^b^	10.17 ± 0.07^b^	9.59 ± 0.22^b^	9.29 ± 0.13^b^

Note

Different letters in each column indicate statistical significant differences in mean values (*p* < .05).

Abbreviations: MUFA, monounsaturated fatty acids; PUFA, polyunsaturated fatty acids; SFA, saturated fatty acids.

The cookies of the F4, F5, and F7 formulations significantly reduced the saturated fat content when compared to the other formulations, besides presenting a significant amount of oleic acid (30.87%, 32.87%, and 33.91%, respectively) in relation to unsaturated fatty acids. Leite, Carrão‐Panizzi, Curti, Dias, and Seibel (2013) obtained oleic acid values of ~29.83% for coconut cookies, values close to those found in this study. In this context, it is feasible to say that cookies formulated with partial substitution of *PA* fat contribute with a significant amount of oleic acid, thus showing a well‐balanced ratio of fatty acids.

### 3.4 Microbiological and sensory analysis

All formulations were microbiologically evaluated, and results are presented in Table 6. The absence of *Salmonella *sp. in 25 g was observed in all formulations. The presence of thermotolerant coliforms was lower than 0.3 NMP/g and the values of *Staphylococcus coagulase* were <10 CFU/g showing that the conditions of handling and processing were adequate, making these foods safe for sensory analysis.

**Table 6:** Microbiological analysis of the cookies formulated containing or not fats of PA and/or PI almond

Cookie formulation	*Salmonella* sp. (cfu/g)	Coliform 45°C (NMP/g)	Staphylococcus coagulase (CFU/g)
F1	NG	0.10	<1.0
F2	NG	0.11	<1.0
F3	NG	0.09	<1.0
F4	NG	0.11	<1.0
F5	NG	0.08	<1.0
F6	NG	0.10	<1.0
F7	NG	0.11	<1.0

Abbreviation: NG, no growth detected.

The panelists' scores for the sensory attributes of the cookies are presented in Table 7. Cookies of all formulations presented scores >6 (slightly liked) for all attributes, all of which were sensorially accepted. However, the F5 formulation (50% of the *PA* fat and 50% of the *PI* almond) presented superior acceptability and sensorial preference, obtaining significantly (*p* ≤ .05) higher scores for all attributes evaluated in comparison with the other formulations. Concerning the intention to purchase, the average scores represented the consumer's intention to “maybe buy/maybe not buy” (note 3), possibly buy (note 4), and certainly buy (note 5). The F5 formulation cookies obtained a score of 5, proving that the partial replacement of hydrogenated vegetable fat with *PA* fat and grated coconut with *PI* almond favored the panelists' purchasing decision.

**Table 7:** Mean values of the panelists' scores for the sensorial attributes of the cookies formulated containing or not fats of *PA* and/or *PI* almond

Parameters	Cookie formulation						
	F1	F2	F3	F4	F5	F6	F7
Aroma	6.63 ± 1.52^a^	6.60 ± 1.35^a^	6.48 ± 1.81^a^	6.97 ± 1.31^a^	7.62 ± 0.82^b^	7.82 ± 0.82^a^	6.63 ± 1.22^a^
Color	6.66 ± 1.65^a^	6.69 ± 1.54^a^	6.81 ± 1.57^a^	7.16 ± 1.44^a^	7.84 ± 1.12^b^	7.31 ± 1.16^a^	6.75 ± 1.50^a^
Taste	6.39 ± 1.99^a^	6.77 ± 1.83^a^	6.74 ± 1.65^a^	7.33 ± 1.28^a^	7.99 ± 0.81^b^	7.52 ± 1.31^a^	6.63 ± 1.47^a^
Sweetness	6.56 ± 1.85^a^	6.68 ± 2.11^a^	6.52 ± 1.99^a^	7.41 ± 1.82^a^	7.82 ± 0.99^b^	7.34 ± 1.25^a^	6.51 ± 1.34^a^
Texture	7.09 ± 1.97^a^	7.04 ± 1.46^a^	7.34 ± 1.50^a^	7.71 ± 1.22^a^	8.52 ± 0.99^b^	7.49 ± 1.27^a^	7.67 ± 1.56^a^
Overall acceptability	6.55 ± 1.97^a^	6.96 ± 1.94^a^	6.72 ± 2.41^a^	7.40 ± 1.30^a^	7.87 ± 0.97^b^	7.58 ± 1.15^a^	6.85 ± 1.31^a^
Buy intention	2.8 ± 0.27^b^	3.4 ± 0.19^b^	2.5 ± 0.11^b^	3.5 ± 0.40^c^	5.1 ± 0.87^a^	3.4 ± 0.22^c^	3.4 ± 0.24^b^

Note

Different letters in each column indicate statistical significant differences in mean values (*p* < .05).

Cookies with total fat replacement with *PA* fat (F1) and coconut with *PI* almond (F3) presented a lower percentage of purchase intention, probably due to the fat perception. The flavor and overall acceptance mean values (Table 5) confirm these results. Therefore, the F5 formulation cookies elaborated with 50% of *PA* fat and 50% of *PI* almond presented better results in all evaluations.

## 4 CONCLUSIONS

Cookies made with *PA* fat and *PI* almond presented high levels of protein, lipids, carbohydrates, and energy. The saturated fatty acids, especially palmitic and lauric acids, were predominant in the lipid profile of the cookies formulated with *PA*. Among the unsaturated fatty acids present, oleic acid had a higher percentage. Cookies formulated with 50% *PA* fat and 50% *PI* almonds (F5) had acceptable sensory and microbiological characteristics. The high content of dietary fiber indicates that this product may be a good source of fibers. The results presented throughout this study indicate that unconventional sources of lipids such as *PA* fat and palm kernel such as *PI* can be used without loss of quality in cookies. The use of these fruits in the increment of new ingredients enables their use in the development of food products while valuing natural resources.

## CONFLICT OF INTEREST

The authors declare no conflict of interest.

## ETHICAL APPROVAL

This study was approved by the Institutional Review Board of Federal University of Grande Dourados (Number: 2.702.611).
